# Supplementary material for: The interactome of intact mitochondria by cross-linking mass spectrometry provides evidence for coexisting respiratory supercomplexes
Source: Mol Cell Proteomics. 2017 Dec 8;17(2):216–32. doi: 10.1074/mcp.RA117.000470 (PMC5795388; doi:10.1074/mcp.RA117.000470)
Supplement: Supplemental Data [file supp_RA117.000470_133922_0_supp_23978_fzffwf.zip › spectra_annotation/mito_spectra_annotation/329-1-6-1-12-1.pdf]

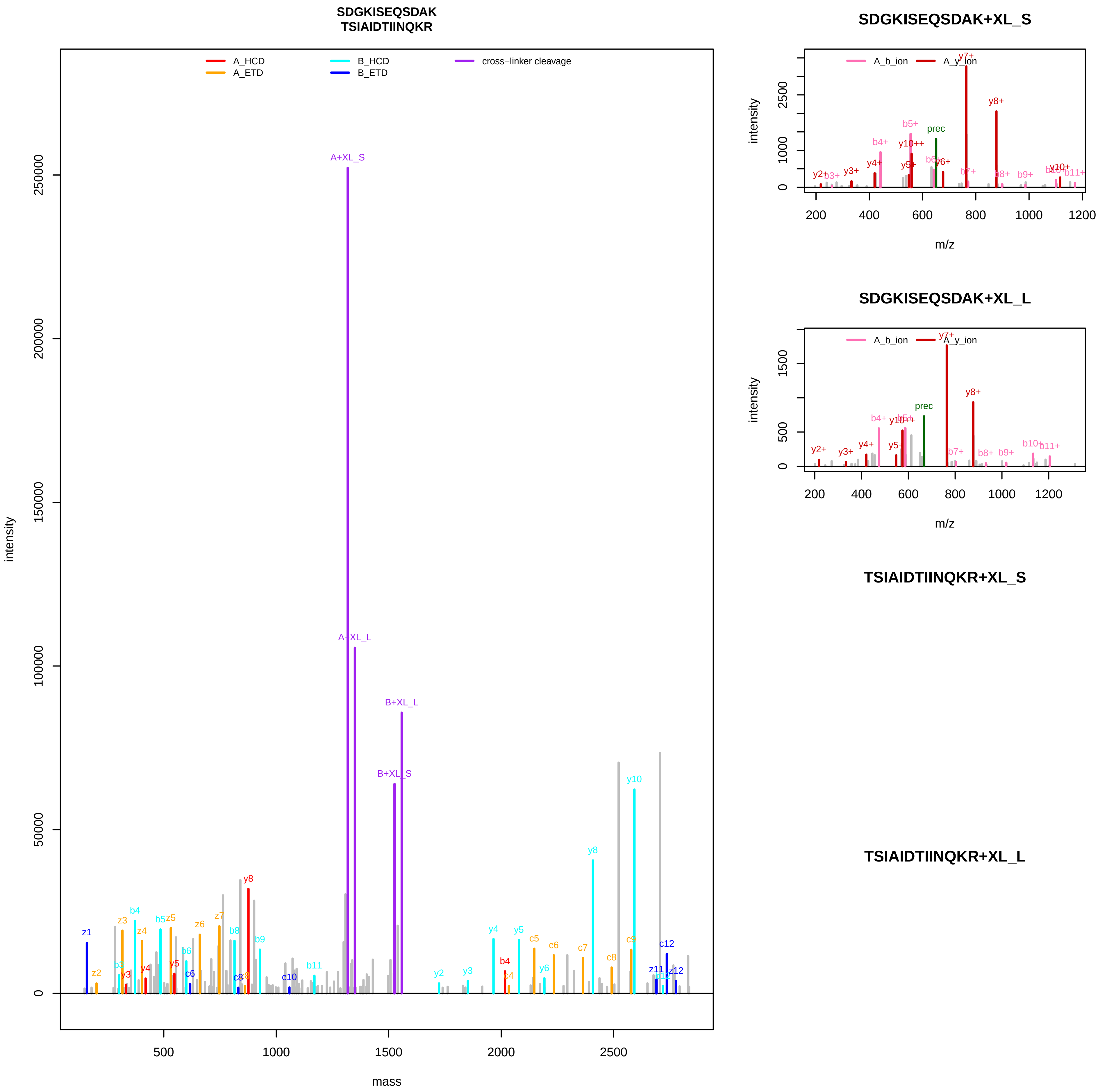
SDGKISEQSDAK
TSIAIDTIINQKR
A_HCD
A_ETD
B_HCD
B_ETD
cross−linker cleavage
A+XL_S
A+XL_L
B+XL_L
B+XL_S
intensity
mass
0
50000
100000
150000
200000
250000
500
1000
1500
2000
2500
z1 z2 b3 z3 y3 b4 z4 y4 b5 z5 y5 b6 z6 c6 z7 b8 c8 y8 b9 c10 b11 y2 y3 y4 y5 b4 c4 c5 y6 c6 c7 y8 c8 c9 y10 z11 c12 z12
SDGKISEQSDAK+XL_S
A_b_ion
A_y_ion
prec
y2+ b3+ y3+ y4+ b4+ b5+ y10++ y5+ b6+ y6+ y7+ b7+ y8+ b8+ b9+ b10+ y10+ b11+
intensity
m/z
0
1000
2500
200
400
600
800
1000
1200
SDGKISEQSDAK+XL_L
A_b_ion
A_y_ion
prec
y2+ y3+ y4+ b4+ y10++ b5+ y5+ y7+ b7+ y8+ b8+ b9+ b10+ b11+
intensity
m/z
0
500
1500
200
400
600
800
1000
1200
TSIAIDTIINQKR+XL_S
TSIAIDTIINQKR+XL_L
